# Multiple immunofluorescence labelling of formalin-fixed paraffin-embedded (FFPE) tissue

**DOI:** 10.1186/1471-2121-9-13

**Published:** 2008-03-19

**Authors:** David Robertson, Kay Savage, Jorge S Reis-Filho, Clare M Isacke

**Affiliations:** 1 Breakthrough Breast Cancer Research Centre, The Institute of Cancer Research, 237 Fulham Road, London SW3 6JB, UK

## Abstract

**Background:**

Investigating the expression of candidate genes in tissue samples usually involves either immunohistochemical labelling of formalin-fixed paraffin-embedded (FFPE) sections or immunofluorescence labelling of cryosections. Although both of these methods provide essential data, both have important limitations as research tools. Consequently, there is a demand in the research community to be able to perform routine, high quality immunofluorescence labelling of FFPE tissues.

**Results:**

We present here a robust optimised method for high resolution immunofluorescence labelling of FFPE tissues, which involves the combination of antigen retrieval, indirect immunofluorescence and confocal laser scanning microscopy. We demonstrate the utility of this method with examples of immunofluorescence labelling of human kidney, human breast and a tissue microarray of invasive human breast cancers. Finally, we demonstrate that stained slides can be stored in the short term at 4°C or in the longer term at -20°C prior to images being collected. This approach has the potential to unlock a large in vivo database for immunofluorescence investigations and has the major advantages over immunohistochemistry in that it provides higher resolution imaging of antigen localization and the ability to label multiple antigens simultaneously.

**Conclusion:**

This method provides a link between the cell biology and pathology communities. For the cell biologist, it will enable them to utilise the vast archive of pathology specimens to advance their *in vitro *data into *in vivo *samples, in particular archival material and tissue microarrays. For the pathologist, it will enable them to utilise multiple antibodies on a single section to characterise particular cell populations or to test multiple biomarkers in limited samples and define with greater accuracy cellular heterogeneity in tissue samples.

## Background

Immunohistochemistry (IHC) is one of the pillars of modern diagnostic pathology and a fundamental research tool in both pathology and translational research laboratories. Currently, labelling of FFPE specimens most commonly involves a biotinylated secondary antibody followed by an avidin-biotin-peroxidase complex and development with a soluble chromogenic substrate. This approach is robust and reliable, and increasingly can be automated for labelling, image acquisition and scoring. However, as a research tool there are three major limitations. First, it is primarily used to reveal one protein at a time; multiple colour approaches by combining peroxidase with other development systems are less than satisfactory and cannot be used to examine the co-localization of two antigens in the same subcellular compartment. Second, the resolution of antigen localization is limited due to the chromogenic substrate precipitate and the thickness (3 – 4 μm) of the sections imaged in the light microscope. Third, chromogenic systems saturate easily, which restricts semi-quantitative analysis. Immunofluorescence labelling, on the other hand, has the capability for multiple labelling and is of higher resolution due to the fluorophores being directly conjugated to the antibody. Nevertheless, immunofluorescence labelling is not often used for FFPE specimens, the perceived mantra being that the inherent autofluorescence of such specimens makes high quality immunofluorescence imaging capricious. This has placed two severe restrictions on investigators. First, it has limited fluorescence imaging to tissue cryosections and hence restricts analysis of clinical material. Second, as cell and tissue preservation is lower in cryosections than in FFPE sections, the quality of morphological findings is frequently compromised. This is particularly the case in tissues that are difficult to cryosection, for example cartilage, bone and those that contain a high fat content such as the breast. In recent years, there have been a number of reports describing the immunofluorescence labelling of FFPE sections [1-10] but, for a variety of reasons, these methods have not been taken up widely by the scientific community (see Discussion). Similarly, quantitative immunofluorescence labelling of FFPE material, particularly that in tissue microarrays, has been achieved by the development of computer assisted fluorescence imaging systems [11]. Although these systems have an important role in translational research, there is still an urgent need for a high resolution method which can be employed by the wider research community. To this end, we have taken a systematic approach to develop a robust protocol for coupling antigen retrieval, indirect immunofluorescence and confocal laser scanning microscopy to image FFPE sections. Using this method, we demonstrate that multicolour immunofluorescence imaging of FFPE material is readily achievable and that this method provides excellent images. Of note, the data shown here were not subjected to any image manipulation.

## Results

**Figure 1:**
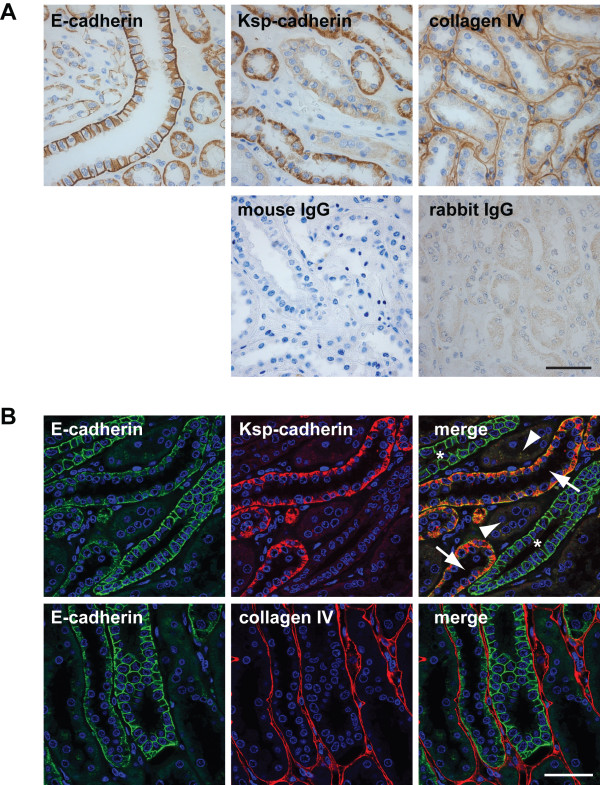
**Expression of E-cadherin, Ksp-cadherin and collagen IV in kidney**. 3 μm FFPE sections of adult human kidney were dewaxed, rehydrated and subject to antigen retrieval. **A**. For immunoperoxidase labelling, sections were incubated with the indicated antibodies and detection was achieved with the Vectastain avidin-biotin complex (ABC) system used according to the manufacturer's protocol (Vector Laboratories). Slides were counterstained with haematoxylin. **B**. Sections were stained as described in the Methods with anti-E-cadherin mAb and anti-Ksp-cadherin mAb or with anti-E-cadherin mAb and anti-collagen IV Ab followed by Alexa488-conjugated goat anti-mouse IgG_2A _and Alexa555 goat anti-mouse IgG_1 _or Alexa488-conjugated goat anti-mouse IgG_2A _and Alexa555 goat anti-rabbit Ig. Nuclei were counterstained with DAPI. Arrows indicate tubules expressing both cadherins, arrowheads indicate tubules expressing neither cadherin, * indicates tubules expressing E-cadherin alone. Scale bar, 50 μm.

To demonstrate the utility of this method, three examples are provided. First, the expression of E-cadherin (cadherin-1), Ksp-cadherin (kidney-specific cadherin, cadherin-16) and collagen IV was examined in human kidney (Figure 1 and Additional file 1). IHC analysis clearly shows a differential labelling of the cadherins in different tubules, each of which is surrounded by a collagen IV containing basal lamina. However, with double-label immunofluorescence the existence of 3 distinct types of tubule (as defined by E- and Ksp-cadherin expression) becomes apparent. In particular, such analysis shows tubules which express neither E- nor Ksp-cadherin. Further, the thin optical sections collected by confocal microscopy demonstrate clearly that this method provides a higher level of detail as to the subcellular distribution of antigens. Control experiments demonstrated the specificity of the secondary antibodies and the absence of background staining [see Additional file 1(2)].

**Figure 2:**
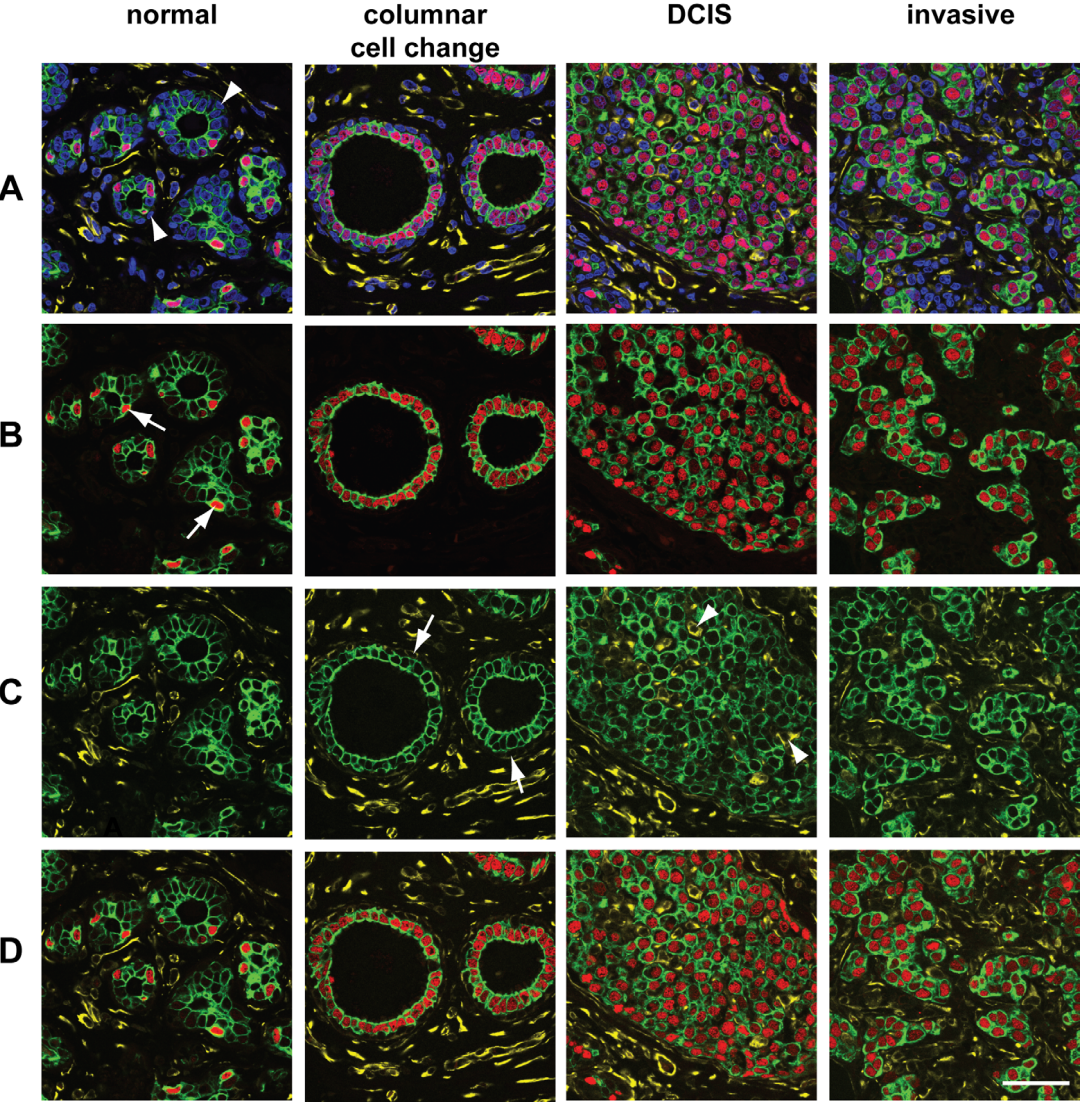
**Expression of cytokeratin 8/18, oestrogen receptor and vimentin in normal human breast and breast cancer**. 3 μm FFPE sections of human adult breast containing both normal tissue and columnar cell change, and an ER-positive breast cancer showing areas of DCIS and invasive ductal carcinoma were stained anti-Ck 8/18 mAb, anti-vimentin mAb and anti-ER Ab followed by Alexa488 goat anti-mouse IgG_1_, Alexa633 goat anti-mouse IgG_2A _and Alexa555 goat anti-rabbit Ig. Nuclei were counterstained with DAPI. In all images colours are as follows: Ck 8/18 (green), ER (red), vimentin (yellow), DAPI (blue). **A**. 4 colour images, arrowheads indicate Ck 8/18-negative myoepithelial cells. **B**. Ck 8/18 and ER, arrows indicate ER expression in luminal epithelial cells with high Ck 8/18 expression in the normal breast. **C**. Ck 8/18 and vimentin, arrows indicate vimentin expression in the myoepithelial cells of columnar cell change, arrowheads indicate vimentin-positive, Ck 8/18-negative cells within the DCIS. **D**. Ck 8/18, ER and vimentin. Scale bar, 50 μm.

In the second example, it is demonstrated that high quality 4 colour fluorescence images can be readily obtained from archival FFPE material; in this case normal breast tissue, breast tissue with columnar cell change and both ductal carcinoma *in situ *(DCIS) and invasive components of an oestrogen receptor (ER) positive breast cancer (Figure 2 and Additional file 1(3)). As expected in the normal breast tissue, ER expression is limited to a subset of cytokeratin 8/18 (Ck 8/18) positive luminal epithelial cells and vimentin expression is restricted to the intra- and interlobular stromal fibroblasts and the vasculature with low level staining in the myoepithelial cells. However, multicolour confocal imaging also reveals that expression of ER in the normal breast is associated with cells expressing high levels of Ck 8/18; that there is an upregulation of vimentin expression in the Ck 8/18 negative myoepithelial cells in the columnar cell change [see also Additional file 1(4)]; and that scattered vimentin-positive, Ck 8/18 negative cells admixed with neoplastic cells can be found in high grade DCIS.

**Figure 3:**
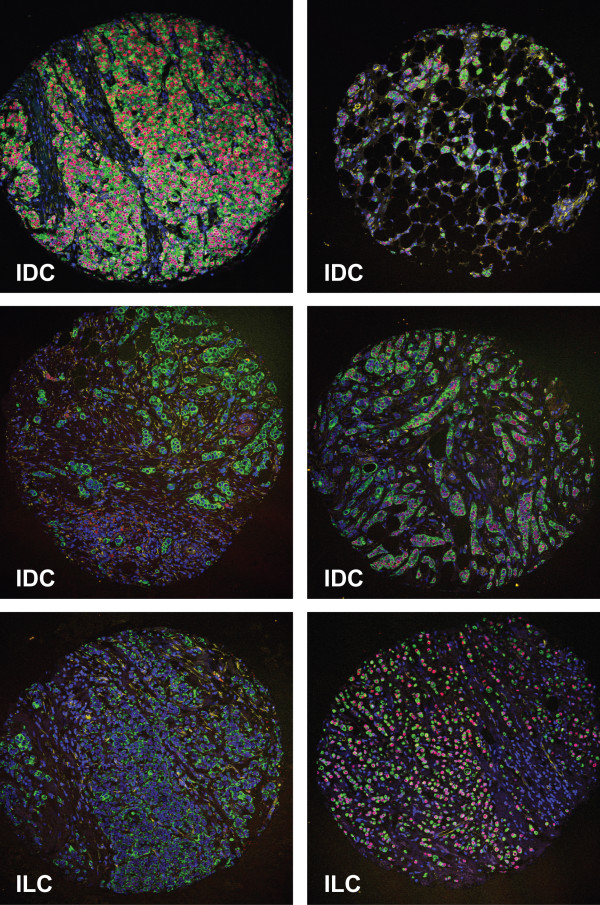
**Immunofluorescence labelling of tissue microarrays slides**. A tissue microarray containing 0.6 mm cores from 245 invasive breast cancers was stained as described in Figure 2. Ck 8/18 (green), ER (red), vimentin (yellow), DAPI (blue). Images from cores of 4 invasive ductal carcinomas (IDC) and 2 invasive lobular carcinomas (ILC) are shown.

In the third example, a tissue microarray containing cores from 245 archival invasive breast cancers [12,13] was stained with the same antibody combination as described above. As illustrated in Figure 3, high quality images can be collected from these samples.

**Figure 4:**
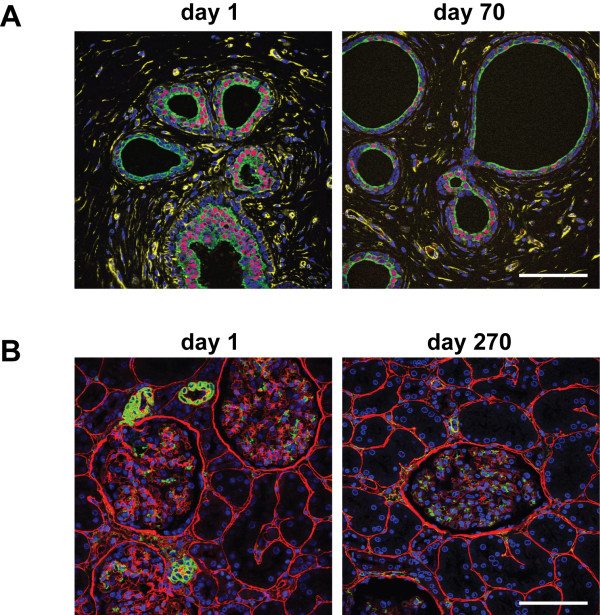
**Storage of FFPE samples after immunofluorescence labelling**. **A**. 3 μm FPPE section of columnar cell change human breast was stained as described in Figure 2. Images were collected directly after labelling (day 1) and again following 70 days storage of the stained slides at 4°C. **B**. 3 μm FFPE section of human kidney was stained with anti-collagen IV Ab (red) and mouse anti-alpha smooth muscle actin mAb (green) followed by Alexa555 goat anti-rabbit Ig and Alexa488-conjugated goat anti-mouse IgG_2A_. Nuclei were counterstained with DAPI (blue). Images were collected directly after labelling (day1) and again following 270 days storage of the stained slides at -20°C. Scale bar, 50 μm.

To date we have successfully employed this method with a range of different primary and secondary antibodies on a large number of freshly fixed and archival samples from different normal tissues and tumour types. One perceived limitation of immunofluorescence labelling is that stained sections can only be stored for a short period of time prior to collecting images. We demonstrate here that stained slides can be stored for at least 10 weeks at 4°C or 9 months at -20°C and examined by confocal microscopy on multiple occasions with only a negligible reduction in the quality of images collected (Figure 4).

## Discussion

We describe here an optimised method for the collection of high quality immunofluorescence images from FFPE sections. The first element that is key to the success of the method is the use of a confocal microscopy has the following advantages over the epifluorescence microscope: (a) the collection of a thin (0.5 μm) optical section, whose thickness is smaller than that of the section (3 – 4 μm), means that the volume of the section contributing to the autofluorescence is less, (b) a conventional mercury vapour lamp in an epifluorescence microscope produces light from 250–800 nm wavelength, all of which has the potential to create autofluorescence. Laser light illumination from the confocal microscope is of a much narrower bandwidth (<5 nm) and thus a significant amount of the visible spectrum light is removed from the excitation process. Furthermore, on the confocal microscope the amount of light from each laser can be adjusted, thus creating the opportunity to minimise the excitation process which in turn reduces autofluorescence, (c) the confocal microscope allows the setting of numerically defined collection windows, which are more precise and efficient than the epifluorescence microscopy dichroic and trichroic glass filters, (d) in the confocal microscope, images can be collected in a sequential mode, which means that at any given time during the collection process only one laser is exciting the tissue and only one collection window is open. This effectively eliminates bleedthrough which can be a significant difficulty in epifluorescence microscopy. The second key issue is that the new generation AlexaFluor dyes are more stable and brighter than the previous generation of fluorescence conjugates such as fluorescein isothiocyanate (FITC), rhodamine, Texas Red, Cy3 and Cy5, and their use can be tailored to the lasers available on different confocal microscopes. The final element of our optimised method is the implementation of careful microscopy practises in which the individual antibodies, the antibody combinations to be used, and the labelling conditions are optimised prior to labelling of important specimens. It is the combination of all these factors that leads to the improvement in image quality described here.

In the past decade, there have been reports of immunofluorescence labelling of FFPE sections from other laboratories [1-10] all of which have made valuable contributions to the field but have not been widely adopted by the scientific community. At least in part, this reflects their use of older generation fluorescence conjugates, epifluorescence microscopes and/or complex image analysis systems. Of particular interest have been reports that autofluorescence can be reduced by the use of reagents such as Sudan Black B [14,15]. As discussed here, the use of the confocal microscope largely reduces the problem of autofluorescence, but it will be important in future studies to test such reagents in the context of this method. Similarly, although we demonstrate here that immunofluorescent labelling of FFPE sections is at least as sensitive as IHC labelling using the Vectastain avidin-biotin complex (ABC) system from Vector Laboratories (Figure 1) or the EnVision+ System-HRP from Dako (data not shown), future studies will be required to assess the sensitivity compared to the new generation of amplification systems such as HRP polymers.

## Conclusion

In conclusion, this method helps to bridge a gap between classical IHC of FFPE material, immunofluorescence labelling of fresh frozen material and high throughput systems for FFPE immunofluorescence detection. The ability to collect routinely high quality multicolour images from FFPE sections using a confocal microscopy combines the advantages of labelling material which is morphologically well preserved with the ability to study co-expression of multiple markers and to detect subcellular antigen localization within tissue samples. It is often difficult to know the orientation of the tissue within a frozen sample and to locate the desired area for analysis. This can only be determined by cutting a cryosection and counterstaining with haematoxylin and eosin. Following trimming and reorientation of the block, this process has to be repeated until the material is correctly positioned, and as a consequence is a time consuming and tedious process. As FFPE blocks routinely have a haematoxylin and eosin stained reference section, these can be used to select the block best suited for the investigation prior to employing the immunofluorescence labelling method described here. Therefore this method can be employed as an efficient research tool to utilise the vast and invaluable archive of FFPE tissues for more detailed cell biology analysis.

## Methods

Full detail of the protocol we have used is provided below, together with suggestions for optimising the method for individual users. Antibodies used in this study are detailed in Table 1.

**Table 1:** List of antibodies, suppliers and concentrations used

**Antigen**	**Isotype**	**Suppliers**	**Concentration**
E-cadherin	mouse IgG_2A_	BD Biosciences, #610181 (Oxford, UK)	0.25 μg/ml
Ksp-cadherin	mouse IgG_1_	Zymed, clone 4H6/F9, #18–7383 (InVitrogen)	0.4 μg/ml
collagen IV	rabbit polyclonal	Acris, #R1041 (Herford, Germany)	5.0 μg/ml
Ck 8/18	mouse IgG_1_	Novocastra, clone 5D3, #5D3CE (Newcastle upon Tyne, UK)	0.94 μg/ml
vimentin	mouse IgG_2A_	Dako, clone Vim 3B4, #M7020	0.25 μg/ml
ER	rabbit polyclonal	Labvision, clone SP1, #RM-9101-S1 (Runcorn, Cheshire, UK)	1:400
αSMA	mouse IgG_2A_	Sigma, #A2547 clone 1A4	0.88 μg/ml

**Second layer antibody**		**Company**	**Concentration**

Alexa488-conjugated goat anti-mouse IgG_2A_		Invitrogen, #A21131	2.0 μg/ml
Alexa488-conjugated goat anti-mouse IgG_1_		Invitrogen, #A21121	2.0 μg/ml
Alexa555-conjugated goat anti-mouse IgG_1_		Invitrogen, #A21127	2.0 μg/ml
Alexa555-conjugated goat anti-rabbit Ig		Invitrogen, #A21429	2.0 μg/ml
Alexa633-conjugated goat anti-mouse IgG_2A_		Invitrogen, #A21136	2.0 μg/ml

### Protocol: Starting material

Starting material is formalin-fixed paraffin embedded (FFPE) tissues. For this, tissue is fixed in 10% neutral buffered formalin (Bios Europe, Lancashire, UK) overnight at room temperature, then processed using a Tissue-Tek VIP automatic tissue processor (Sakura, Finland) with a standard 14 hour protocol and embedded into paraffin wax (Tissue-Tek). 3–4 μm sections are cut from the embedded blocks and floated onto a warm (42°C) water bath from where they are picked up onto Superfrost plus slides (Cat No 631–0108, VWR International, Lutterworth, Leicestershire, UK). Slides are placed in a vertical rack and dried overnight at 37°C in a fan assisted cabinet. The next day, slides are dated and placed in a container which is then purged with oxygen-free nitrogen gas (BOC gases, Guildford, Surrey, UK). The containers are stored at 4°C. To carry out a labelling experiment, the required slides are removed and the container with remaining slides is again purged with oxygen-free nitrogen gas before replacing at 4°C.

### Notes on slide storage

DiVito and colleagues [16] have investigated the long term preservation of antigenicity on tissue microarrays, and their preferred method of storage is to recoat the cut sections with paraffin wax and store the slides under nitrogen gas. We adopted this method, but did not find any easily discernible difference in image quality when labelling slides that have been left uncoated on the bench for up to a month compared to those that have been wax coated and stored under nitrogen gas (data not shown). At present, we routinely store cut sections uncoated under nitrogen gas, but we would advise individual investigators to test the optimal storage conditions for their samples and antibodies.

### Protocol: Dewaxing and antigen retrieval

Slides are loaded into glass slide holders and dewaxed as follows: Twice in 100% xylene (Fisher Scientific, Loughborough, Leicestershire, UK) for 5 minutes with 10 seconds agitation every 30 seconds, 100% ethanol (BDH, Poole, Dorset, UK) with 10–20 seconds agitation, once in 90% ethanol with 10–20 seconds agitation, once with 70% ethanol with 10–20 seconds agitation and twice in H_2_O with 10–20 seconds agitation. The rack is transferred into 200 ml of pre-warmed (94°C–96°C) Dako (Ely, Cambridgeshire, UK) target retrieval solution (S1699, 20 ml of stock into 180 ml H_2_O) in a glass container in the water bath which is set at 95°C. Antigen retrieval is 30 minutes in the water bath, 20 minutes on the bench and 5 minutes in running water.

### Notes on dewaxing and antigen retrieval

It is essential that the slides are completely dewaxed before labelling. For some uncoated slides, xylene treatment may have to be increased to 2 × 10 minutes. For slides that have been recoated in wax, we recommend 2 × 20 minutes incubation in xylene. As with immunohistochemical labelling, it is also important for each investigator to first establish the antigen retrieval method best suited to the combination of antibodies to be used. In the experiments described here, robust labelling with all antibodies is achieved using the Dako target retrieval solution.

### Protocol: Immunofluorescence labelling

Slides are removed from the running water and wiped around the section to create an 'island' onto which 100 – 200 μl of phosphate buffered saline (PBS) is carefully added to prevent the section from drying out. Next an ImmEdge pen (H-4000, Vector Laboratories, Peterborough, UK) is used to 'ring' the 'island'. The slide is shaken to remove excess PBS and 200 μl of immunofluorescence buffer (IFF) (PBS plus 1% bovine serum albumin (A3059, Sigma, Poole, Dorset, UK) and 2% foetal calf serum (Invitrogen, Paisley, UK), filtered through a 0.2 μm filter) is carefully pipetted onto the section before placing the slides in a moist chamber at room temperature. All following incubations are at room temperature with gentle mixing on a rocking platform, except where the primary antibody is applied overnight in which case the incubation is at 4°C (without rocking). Details of all antibodies used are in Table 1. Primary antibody(s) diluted in IFF for 60 minutes (or overnight at 4°C), 3 × 5 minute washes in PBS, 2 μg/ml secondary antibody diluted in IFF for 60 minutes, 3 × 5 minute washes in PBS containing 1.43 μM 4',6-diamidino-2-phenylindole (DAPI; D21490, Invitrogen,). The volumes used throughout are 100 – 200 μl depending on the size of the section.

Slides are rinsed in PBS, mounted in Vectashield (H-1000, Vector Laboratories) and sealed with clear nail varnish. This is best achieved by placing 8 μl of Vectashield on a 22 × 40 mm coverslip (0.155 – 0.185 mm thickness, VWR International), lowering the coverslip onto the section and gently squeezing out the excess Vectashield before sealing with nail varnish. Stained slides are stored at 4°C. We routinely collect images from slides within 0–5 days after labelling. High quality images can be collected following storage at 4°C for up to 10 weeks (see Figure 4A). However, if it is envisaged that the slides will be examined at later times, we strongly recommend storage at -20°C. As shown in Figure 4B, we have collected images following storage of slides at -20°C for 9 months with minimal loss in quality. Our recommendation is to collect images from slides that have been stored at -20°C within 1 – 4 weeks.

### Notes on antibody concentration, multiple labelling and controls

In the majority of experiments, AlexaFluor-conjugated secondary antibodies (Molecular Probes, Invitrogen) were used. However, successful results have also been obtained with FITC-conjugated antibodies (data not shown).

As with immunofluorescence labelling of fresh or paraformaldehyde fixed samples, optimal results are obtained by first determining the optimal antibody concentration to be used. We routinely do this by titrating the antibody in double dilutions (e.g. 1:50, 1:100, 1:200 and so forth) and finding the lowest dilution that gives strong specific labelling with the low background labelling (i.e. the optimal signal:noise ratio). In conjunction, we check that our routine 60 min labelling at room temperature is optimal for each antibody. If necessary, a longer labelling period and/or labelling at 4°C can be used. In this study all of the secondary antibodies employed were AlexaFluor conjugates supplied as a stock concentration of 2 mg/ml. Doubling dilution experiments on FFPE sections indicates that 2 μg/ml is the optimal concentration for all secondary antibodies. In these optimisation experiments, it is of paramount importance to have predefined positive and negative controls.

The order in which primary and secondary antibodies are added depends on the antibody combinations to be employed. In the examples shown in this manuscript, primary antibodies are mixed together at the appropriate dilution in IFF and added to the section. Following the PBS washes, the different secondary antibodies (each at 2 μg/ml) are added together to the sections. In other experiments, we have successfully performed sequential labelling; for example, incubation of sections with primary mouse monoclonal antibody (mAb), wash in PBS, incubation with Alexa555-conjugated anti-mouse Ig secondary antibody, wash in PBS, incubation with FITC-conjugated mouse mAb (data not shown). Whatever labelling combination is employed, it is essential to conduct control experiments to ensure that there is no antibody cross reactivity. For example, in the experiments shown in Figure 1B, a mouse mAb IgG_2A _is used to detect E-cadherin and a mouse mAb IgG_1 _is used to detect Ksp-cadherin. As control experiments [see Additional file 1(2)], each primary mAb is replaced in turn with the same concentration of antibody from the same species (in this case mouse IgG) to confirm that the anti-mouse IgG_1 _secondary antibody does not recognise the primary IgG_2A _mAb and that the anti-mouse IgG_2A _secondary antibody does not recognise the primary IgG_1 _mAb.

### Protocol: Confocal microscopy

The stained sections are visualised with a Leica SP2 confocal scanning microscope (Leica Microsystems, Milton Keynes, Bucks, UK) set up with the laser outputs controlled via the Acousto-Optical Tunable Filter and the four collection windows using the Acousto-Optical Beam Splitter as follows; 403 nm laser (25%) window 410–483 nm, 488 nm laser (25%) window 493–538 nm, 543 nm laser (100%) window 548–628 nm and 633 nm laser (25%) window 638–700 nm. Images are collected using the microscope in sequential mode with a line average of 4 and a format of 1024 × 1024 pixels. We routinely collect images using a ×20 dry lens (lens specification, HCPLAPOCS NA 0.7; Leica) at ×1 zoom or with a ×40 oil immersion lens (lens specification, HCXPLAPO NA 1.25; Leica) with immersion oil (518F, Zeiss, Welwyn Garden City, Hertfordshire. UK) at ×1 or × 2 zoom. Images are exported from the Leica confocal software into Adobe Photoshop CS2 v.9. In the figures shown in this manuscript, there was no image manipulation performed prior to export into Adobe Illustrator CS2 v12 to generate the figures.

## Abbreviations

Ck, cytokeratin; DAPI, 4',6-diamidino-2-phenylindole; DCIS, ductal carcinoma *in situ*; ER, oestrogen receptor; FFPE, formal-fixed paraffin-embedded; Ig, immunoglobulin; IHC, immunohistochemistry; FITC, fluorescein isothiocyanate; IFF, immunofluorescence buffer; mAb, monoclonal antibody; PBS, phosphate buffered saline; SMA, smooth muscle actin.

## Authors' contributions

DR developed the method, performed all of the immunofluorescence labelling and drafted the manuscript. KS cut the sections and performed all of the immunohistochemistry. JSR-F was involved in the design of the experiments, selected the samples to be analysed, performed the histopathological analysis of samples and participated in writing of the manuscript. CMI was the project leader, participated in the design of experiment and prepared the manuscript. All authors read and approved the final manuscript.

## Supplementary Material

Additional file 1 Additional file 1 contains images equivalent to those shown in Figure 1 but taken at low power. Click here for file
