# Supplementary material for: Multiple immunofluorescence labelling of formalin-fixed paraffin-embedded (FFPE) tissue
Source: BMC Cell Biol. 2008 Mar 19;9:13. doi: 10.1186/1471-2121-9-13 (PMC2288605; doi:10.1186/1471-2121-9-13)
Supplement: Additional file 1 — contains images equivalent to those shown in Figure 1 but taken at low power. [file 1471-2121-9-13-S1.pdf]

Additional file 1 (Robertson et al.)

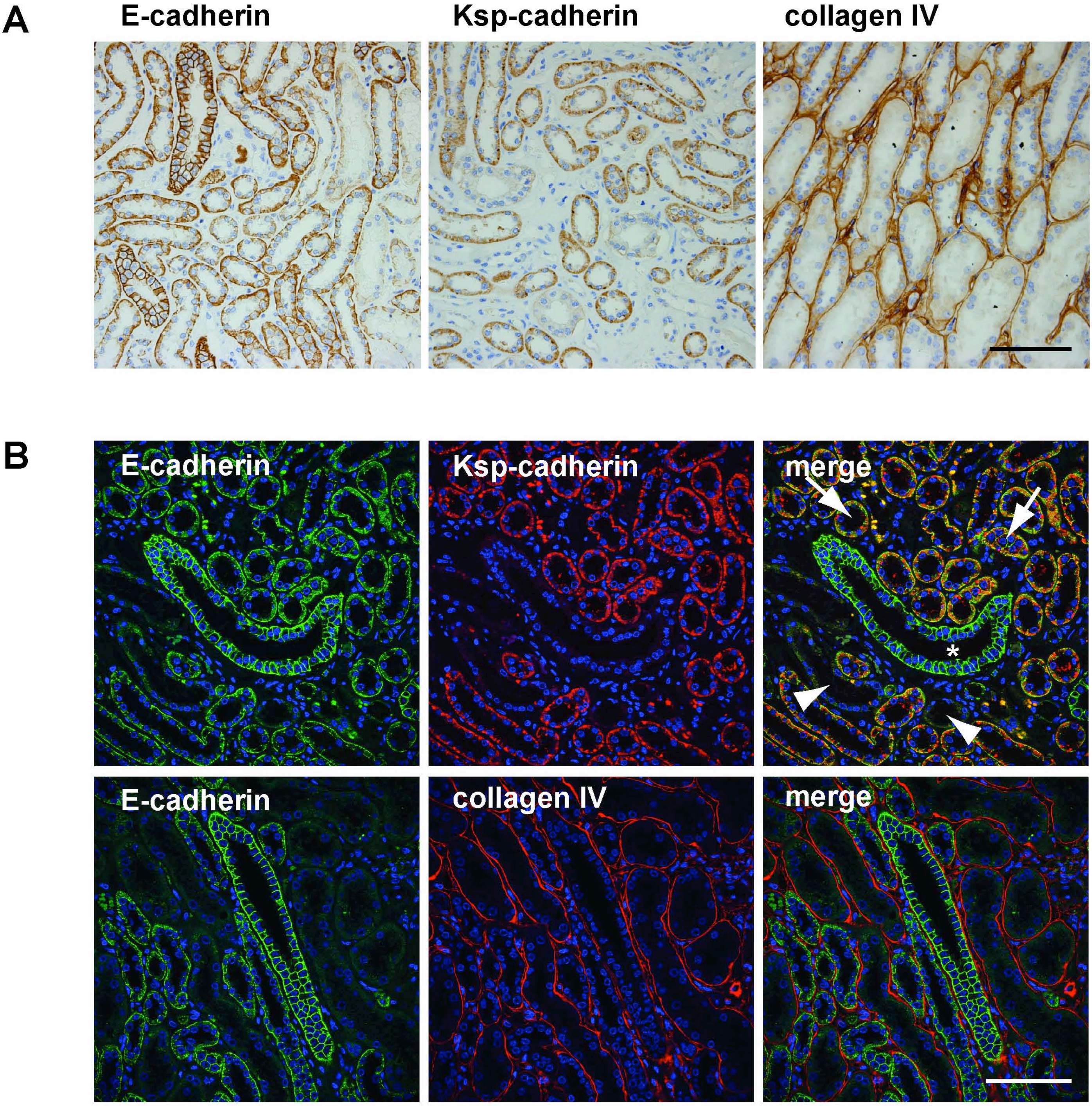

Expression of E-cadherin, Ksp-cadherin and collagen IV in kidney
Low power images from the slides shown in Figure 1. Confocal images were collected using a x20 lens sequentially in three channels. Arrows indicate tubules expressing both cadherins, arrowheads indicate tubules expressing neither cadherin, * indicates tubules expressing E-cadherin alone. Scale bar, 100 µm.

Additional file 2 (Robertson et al.)

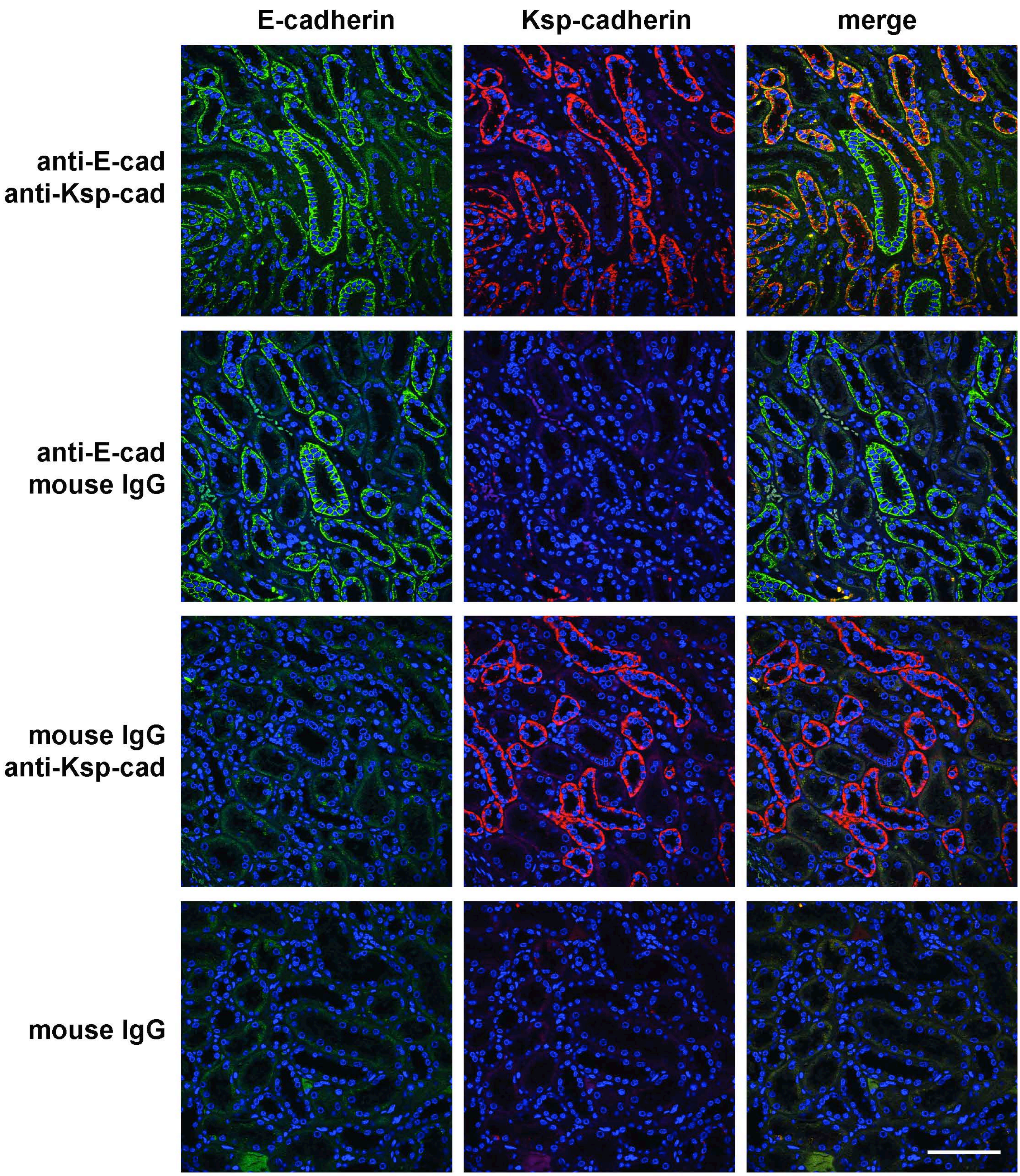

Confocal microscopy control staining

3 µm FFPE sections of adult human kidney were stained as described in Figure 1. As controls, primary antibodies alone or together were substituted with 2 µg/ml mouse IgG. Confocal images were collected sequentially in 3 channels at the same setting for each sample. Images show no cross-reactivity of secondary antibodies or background staining of the secondary antibodies. Scale bar, 100 µm.

Additional file 3 (Robertson et al.)

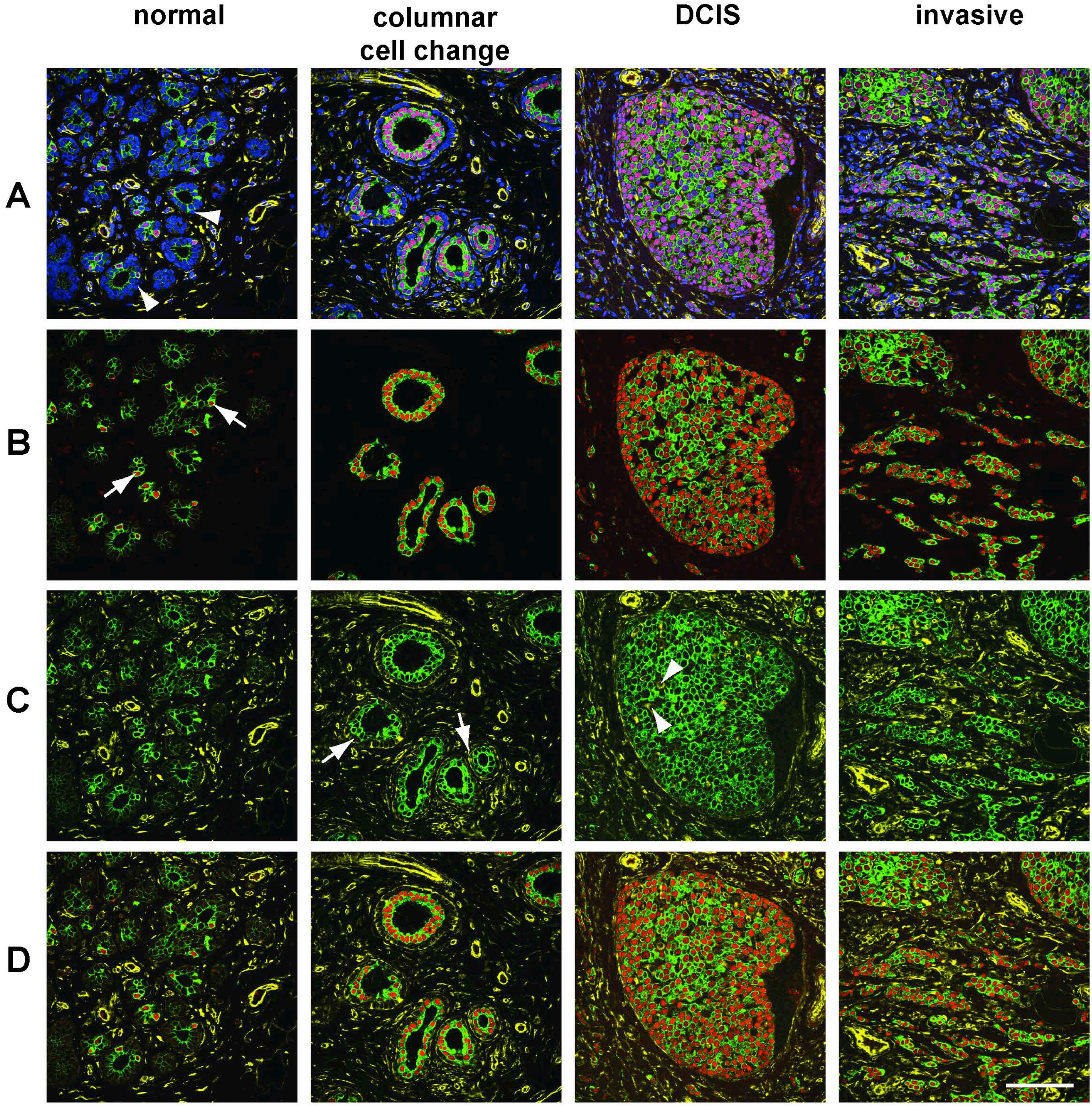

Expression of cyokeratin 8/18, oestrogen receptor and vimentin in normal human breast and breast cancer.
Low power images from the slides shown in Figure 2. Confocal images were collected using a x20 lens sequentially in four channels. In all images colours are as follows: Ck 8/18 (green), ER (red), vimentin (yellow), DAPI (blue). **A.** 4 colour images, arrowheads indicate Ck 8/18-negative myoepithelial cells. **B.** Ck 8/18 and ER, arrows indicate ER expression in luminal epithelial cells with high Ck 8/18 expression positive cells in the normal breast. **C.** Ck 8/18 and vimentin, arrows indicate a low level of vimentin expression in the myoepithelial cells of columnar cell change, arrowheads indicate vimentin-positive, Ck 8/18-negative cells within areas of DCIS. **D.** Ck 8/18, ER and vimentin. Scale bar, 100 μm.

Additional file 4 (Robertson et al.)

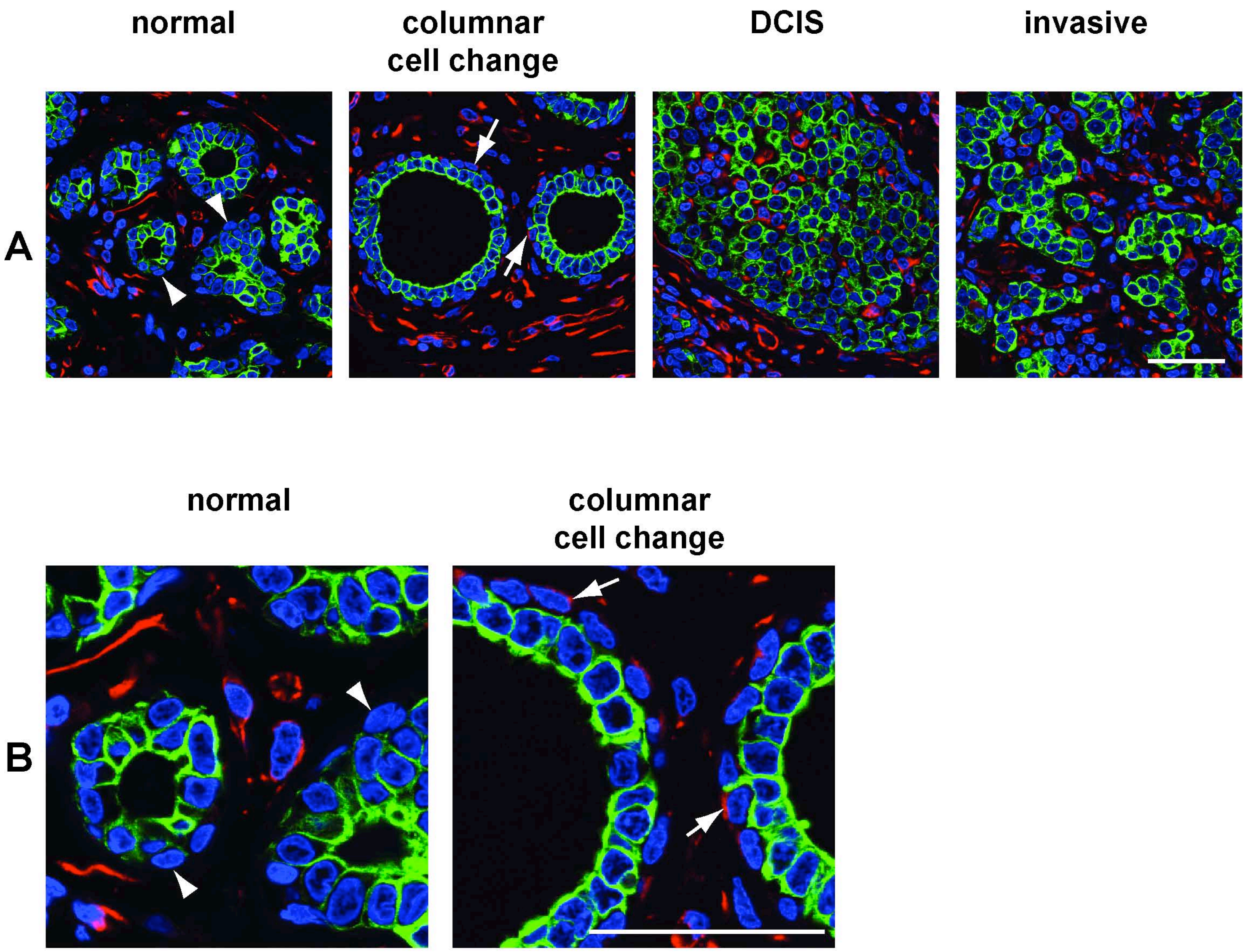

Upregulation of vimentin expression in the myoepithelial cells of columnar cell change. Images identical to those shown in Figure 2 except that Ck 8/18 is shown in green, vimentin in red and DAPI in blue. Arrowheads indicated Ck 8/18-negative, vimentin-negative myoepithelial cells in the normal breast. Arrows indicate Ck 8/18-negative, vimentin-positive myoepithelial cells in columnar cell change. **A**, low power images, **B**, higher power images of selected regions on normal breast and columnar cell change. Scale bar, 50 μm.
